# Latest Advancements in Micro Nano Molding Technologies—Process Developments and Optimization, Materials, Applications, Key Enabling Technologies

**DOI:** 10.3390/mi13040609

**Published:** 2022-04-13

**Authors:** Guido Tosello

**Affiliations:** Department of Mechanical Engineering, Technical University of Denmark, Produktionstorvet, Building 427A, 2800 Kongens Lyngby, Denmark; guto@mek.dtu.dk; Tel.: +45-45-25-4893

Micro and nano molding technologies are continuously being developed due to enduring trends such as increasing miniaturization and the higher functional integration of products, devices and systems. Furthermore, with the introduction of high-engineering-performance polymers, feedstocks and composites, new opportunities in terms of materials properties can be exploited, and consequently, more micro product and micro/nano structured surfaces are currently being designed and manufactured.

Innovations in micro and nano molding techniques are seen in different processes employed in production (e.g., injection molding, micro injection molding, powder micro molding, two-component molding, compression molding, hot embossing, nanoimprint lithography); in the use of new and functional materials including, e.g., nanocomposites; for an ever-increasing number of applications (health-care devices, micro implants, micro analytics systems, mobility and communications products, optical elements, micro electromechanical systems, sensors, micro molded interconnected devices, etc.); in several key enabling technologies that support the successful realization of micro and nano molding processes (micro and nano tooling technologies, process monitoring techniques, micro and nano metrology methods for quality control, simulation, rapid prototyping techniques for micro product development, etc.) and their integration into new manufacturing process chains.

Accordingly, this Special Issue seeks to showcase research papers focusing on the latest developments in micro and nano scale manufacturing using molding techniques as well as their related key enabling technologies to produce both micro products and micro/nano structured surfaces.

The Special Issues consists of 10 original research papers and 2 review papers, which cover fundamental molding process technology development, key enabling technologies, as well as the design and application of these technologies for the fabrication of micro/nano devices and micro structured components.

The papers included in the Special Issue address research, development and recent advancements in four main domains of micro/nano molding: (1) process technology developments and characterization; (2) modeling and simulation; (3) tooling technologies and micro tool design; (4) applications.

(1) Process technology developments and characterization. Calaon et al. [1] analyzed and compared the process capability and design of conventional injection molding and of micro injection molding machines; Wöhner et al. [2] characterized the formation of blisters in film micro insert molding; Loaldi et al. [3] integrated direct ink writing with injection molding to generate micro conductive tracks in polymer devices.
(2) Modeling and simulation. Weng et al. [4] modeled the demolding phase in nano scale molding by using molecular dynamic simulations; Loaldi et al. [5] simulated the micro injection molding process of both three-dimensional micro parts and micro structured components by applying multi-scale meshing and virtual design of experiment techniques.
(3) Tooling technologies and micro tool design. Wang et al. [6] developed the air-shielding electrochemical micromachining (AS-EMM) process to improve the generation of microstructures on metal surfaces; Li et al. [7] analyzed the effects of machining errors on the optical performance of optical aspheric components in ultra-precision diamond turning; Tucker et al. [8] characterized different venting design solutions in micro molding tools and the influence of micro injection molding process parameters on air traps and adiabatic heating.
(4) Applications. Kim et al. [9] presented the production of a miniaturized out-of-plane compliant bistable mechanism (OBM) via micro injection molding; Wu et al. [10] reviewed the state of the art and perspectives on silicon waveguide crossings and discussed the use of polymers as vertical directional coupler materials; Cheng et al. [11] reviewed the design and emerging trends of grating couplers on silicon photonics and presented the possibility of a polymer-based micro packaging application of plasmonic surfaces; Takehara et al. [12] fabricated microneedle arrays in Poly (L-lactic Acid) via micro hot embossing/compression molding and studied the effect of the thermal history on the material crystallinity during the process.

We wish to thank all of the authors who submitted their papers to this Special Issue, entitled “Latest Advancements in Micro Nano Molding Technologies—Process Developments and Optimization, Materials, Applications, Key Enabling Technologies”. We would also like to acknowledge all of the reviewers whose careful and timely reviews ensured the high quality of this Special Issue.

The support and funding from the European Commission Horizon2020 Framework Programme for Research and Innovation through the ProSurf project (“High Precision Process Chains for the Mass Production of Functional Structured Surfaces”, http://www.prosurf-project.eu/, accessed on 5 April 2022, 2018–2021, Project ID: 767589), as well as the Marie Skłodowska-Curie Action Innovative Training Networks MICROMAN (“Process Fingerprint for Zero-defect Net-shape MICROMANufacturing”, http://www.microman.mek.dtu.dk/, accessed on 5 April 2022, 2015–2019, Project ID: 674801) and DIGIMAN4.0 (“DIGItal MANufacturing Technologies for Zero-defect Industry 4.0 Production”, http://www.digiman4-0.mek.dtu, accessed on 5 April 2022, 2019–2024, Project ID: 814225), is acknowledged. The support and funding from the Danish Innovation Fund (https://innovationsfonden.dk/en, accessed on 5 April 2022), through the research projects MADE DIGITAL, Manufacturing Academy of Denmark (http://en.made.dk/, accessed on 5 April 2022, 2017–2020, Project ID: 6151-00006B), Work Package WP3 “Digital manufacturing processes” and QRprod (“QR coding in high-speed production of plastic products and medical tablets”, 2016–2019 Project ID: 5163-00001B) is acknowledged.
